# The association between dietary creatine intake and cancer in U.S. adults: insights from NHANES 2007–2018

**DOI:** 10.3389/fnut.2024.1460057

**Published:** 2025-01-10

**Authors:** Junhui Jiang, Hu Zhao, Jiong Chen, Junhao Du, Weixiang Ni, Baohua Zheng, Junhong Wu, Chunhong Xiao

**Affiliations:** ^1^Fuzong Clinical Medical College of Fujian Medical University, Fuzhou, China; ^2^Department of General Surgery, The 900th Hospital of Joint Logistic Support Force, PLA, Fuzhou, China

**Keywords:** creatine, cancer, age, NHANES, nutrition

## Abstract

**Background:**

Creatine has anti-inflammatory, antioxidant, and immunomodulatory effects. However, its impact on tumors remains uncertain.

**Methods:**

This study used data from the National Health and Nutrition Examination Survey (NHANES) from 2007 to 2018 to investigate the relationship between dietary creatine intake and cancer in American adults. A total of 25,879 participants aged 20 years and older were included, and their medical information, dietary creatine intake, and covariates were collected. Multiple logistic regression models were used to assess the relationships between age, dietary creatine intake, and cancer risk. Restricted cubic spline (RCS) analysis explored the nonlinear relationships between dietary creatine intake, age, and cancer prevalence.

**Results:**

RCS analysis revealed a linear, negative association between dietary creatine intake and cancer risk. For each standard deviation (SD) increase in dietary creatine intake, cancer risk decreased by 5% (adjusted odds ratio (OR) = 0.95, 95% CI: 0.91–0.99, *p* = 0.025). This negative association was strongest among males (adjusted OR = 0.93, 95% CI: 0.88–0.99, *p* = 0.021) and overweight participants (adjusted OR = 0.92, 95% CI: 0.84–0.99, *p* = 0.044). Interaction results indicated specific age group effects. Further analysis showed that higher dietary creatine intake was significantly inversely associated with cancer risk among older adults (adjusted OR = 0.86, 95% CI: 0.77–0.97, *p* = 0.014). RCS analysis revealed a linear, positive correlation between age and cancer risk. For each SD increase in age, cancer risk increased by 3.27 times (adjusted OR = 3.27, 95% CI: 3.07–3.48, *p* < 0.001).

**Conclusion:**

These findings suggest that higher dietary creatine intake may reduce cancer risk in a nationally representative adult population. Further prospective studies are needed to clarify the relationship between dietary creatine intake and cancer risk.

## 1 Introduction

Cancer remains the second leading cause of death worldwide. In the United States alone, projections estimate over 2 million new cancer cases in 2024, approximately 2,001,140 cases, along with an estimated 611,720 cancer-related deaths (1). While some studies suggest that bacterial infections may now rank second after cardiovascular diseases as a leading cause of death, the increasing number of cancer diagnoses and fatalities underscores its profound impact on global health (2). The mechanisms underlying tumorigenesis are highly intricate. Research shows that factors such as smoking, alcohol consumption, and obesity contribute to cancer development by generating carcinogens, damaging normal cell DNA, causing gene mutations, and inducing oxidative stress and immune suppression (2–6).

**Figure 1:**
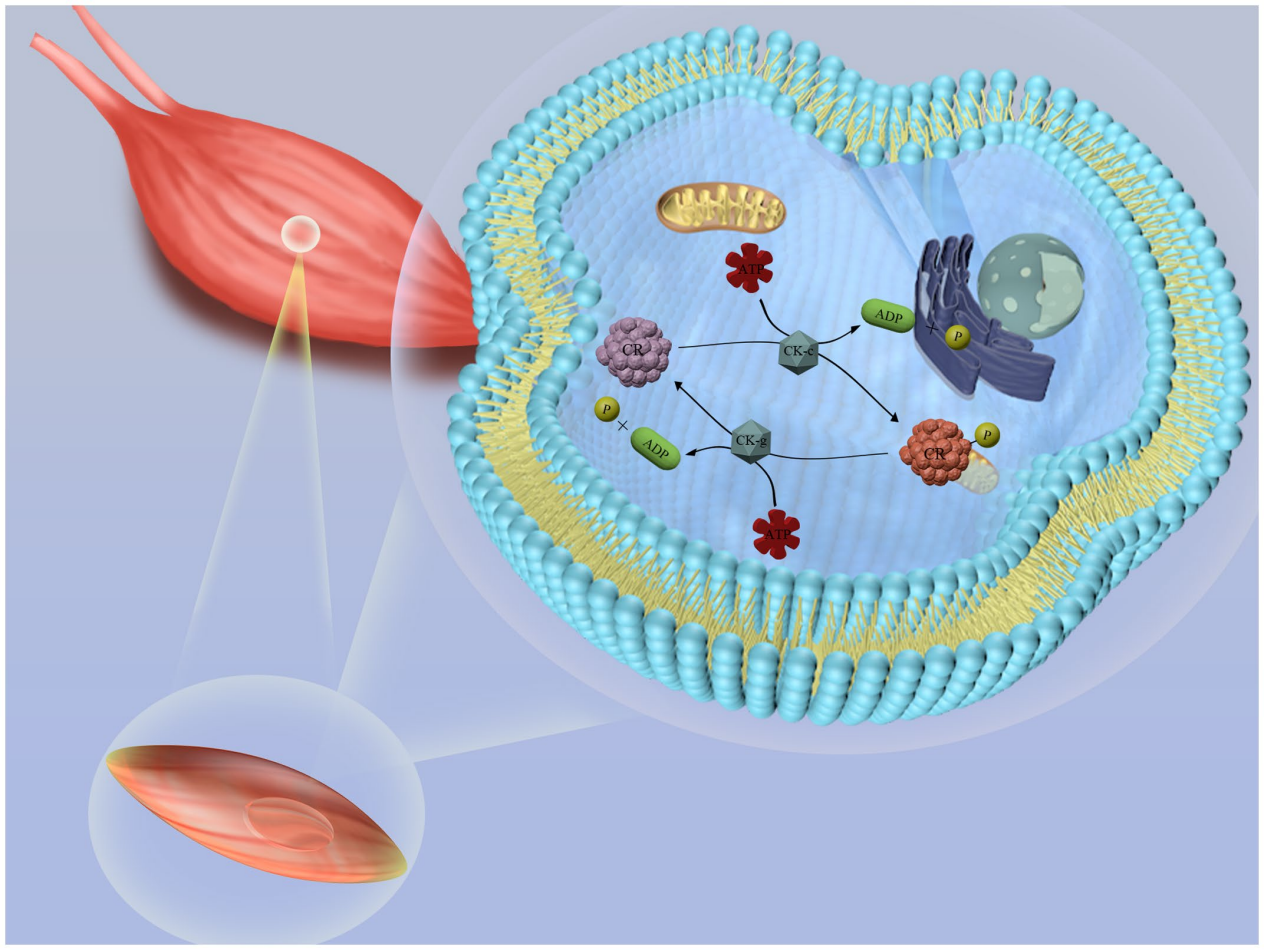
Schematic diagram of creatine metabolism in the body. Creatine reacts with ATP (adenosine triphosphate) in the liver, kidneys, and pancreas to form phosphocreatine (PCr), which is stored in muscle cells. When rapid energy release is required, phosphocreatine decomposes quickly, releasing a phosphate group and regenerating ATP. This newly formed ATP can be immediately utilized by muscle cells to support muscle contraction and other energy-demanding biological processes.

Creatine, an organic acid naturally produced in the body, plays a vital role in energy metabolism within muscle cells (7). Inside the cells, creatine is converted into phosphocreatine (PCr) and stored in muscles, where it provides rapid energy for short-term, high-intensity activities (Figure 1). Due to its performance-enhancing effects, many athletes use creatine supplements (8, 9). In healthy adults, about 2 g of creatine, roughly 1.7% of the total, is excreted daily as creatinine in urine, necessitating the consumption of about 500 g of raw meat per day to maintain optimal levels.

Beyond enhancing athletic performance, creatine also boosts phosphocreatine reserves in brain cells, which promotes brain health and may potentially prevent neurological disorders. A two-year randomized clinical trial on Parkinson’s disease (PD) patients found that those who took creatine monohydrate supplements experienced a notable reduction in depressive symptoms (10). Additionally, emerging studies suggest that creatine could be an effective treatment for depression (11). Moreover, creatine acts as an antioxidant, protecting tissues from oxidative damage and modulating immune responses by influencing macrophage polarization (5, 12, 13).

The relationship between dietary creatine intake and cancer risk remains uncertain. While oxidative stress and immune dysfunction are major drivers of tumorigenesis, creatine’s anti-inflammatory, antioxidant, and immune-modulating properties could counteract these processes. A comprehensive review on creatine and cancer suggests that creatine might suppress tumor growth by enhancing CD8+ T cell antitumor activity (7, 14–18). However, when creatine is converted to phosphocreatine, it may supply ATP to cancer cells, potentially aiding metastasis and invasion (7, 19). Therefore, further investigation is required to clarify the potential connection between creatine and cancer.

This study focuses on a large population-based sample from the United States to determine the association between dietary creatine intake and cancer risk within North American diets. Using data from the National Health and Nutrition Examination Survey (NHANES) conducted by the Centers for Disease Control and Prevention, we aimed to (1) assess cancer prevalence among U.S. community-dwelling adults across quartiles of creatine intake, (2) analyze the association between average daily dietary creatine intake and cancer risk, and (3) examine how sex, age, and BMI might modify this association.

## 2 Materials and methods

### 2.1 Study population

**Figure 2:**
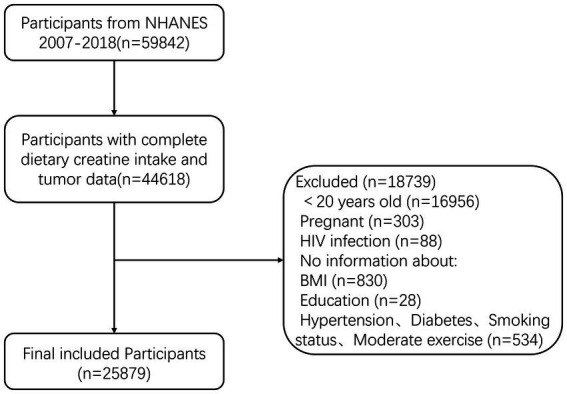
Flowchart of study participant inclusion and exclusion criteria.

The National Health and Nutrition Examination Survey (NHANES), conducted by the National Center for Health Statistics (NCHS), measures the health and nutritional status of adults and children in the non-institutionalized civilian population of the United States. This study used data from the NHANES program for the years 2007–2018, including adult participants aged 20–80+ years. A total of 59,842 participants were initially included, of which 44,618 had complete information on dietary creatine intake and cancer. Exclusions were made for 18,739 participants based on the following criteria: (1) age < 20 years (*n* = 16,956); (2) pregnancy (*n* = 303); (3) HIV infection (*n* = 88); (4) missing BMI (*n* = 830); (5) unspecified educational level (*n* = 28); and (6) incomplete information on other variables (*n* = 534). Ultimately, 25,879 participants were included in the final analysis (Figure 2).

### 2.2 Dietary creatine and cancer

Animal proteins, including meat, poultry, fish, and shellfish, are primary sources of creatine in human diets. Data on individual dietary intake of these proteins were collected from NHANES Day 1 and Day 2 dietary interview questionnaires, which Participants’ total dietary intake was recorded over two distinct 24-h periods, with the initial interview conducted in person during the MEC assessment and the follow-up by phone a few days later. Creatine intake from meat and fish was categorized using USDA food codes into subgroups of the MyPyramid Equivalents database: meat, offal, sausages and cold cuts, poultry, fish and seafood high in *n*−3 fatty acids, and seafood low in *n*−3 fatty acids. The participants’ daily food intake was categorized. The average creatine concentration for all animal protein sources was estimated at 0.11 g/ounce (11, 20). The two-day average creatine intake, calculated in grams, served as the primary exposure for analysis. Cancer was determined for respondents who answered positively to the question, “Have you ever been told by a doctor or other health professional that you had cancer or a malignancy of any kind?”

### 2.3 Standard deviation transformation

Since age and creatine were included as continuous variables in the model, their clinical significance may not be readily interpretable despite statistical significance. Therefore, in this study, age and dietary creatine intake were transformed using standard deviation to provide clinically interpretable insights.

### 2.4 Covariates

Based on a review of relevant literature and clinical expertise, potential covariates included age, sex, race (Mexican American, Other Hispanic, Non-Hispanic and Other race), education level (<9th grade, 9–11th grade, High school diploma/GED, Some College/AA degree, ≥ College graduate), poverty income ratio (PIR), body mass index (BMI), smoking status, alcohol consumption, moderate physical activity, hypertension, diabetes, depression, and blood concentrations of cadmium and lead. Hypertension was defined based on responses to “Ever told you had hypertension” and “taking hypertension prescription.” Diabetes was diagnosed according to “Doctor told you have diabetes,” “Glycohemoglobin >6.5% or Fasting Glucose >7.0 mmol/L or Two Hour Glucose (OGTT) ≥11.1 mmol/L.” Smoking status was categorized as never smoker (smoked fewer than 100 cigarettes in a lifetime), current smoker (currently smoking or smoked more than 100 cigarettes in a lifetime), and former smoker (quit smoking and smoked more than 100 cigarettes in a lifetime). Alcohol consumption history was determined by “Had at least 12 alcohol drinks/lifetime?” and missing data were replaced with “NA.” Depression was screened using the Patient Health Questionnaire (PHQ-9), with scores ranging from 0 to 27, categorized into mild (5), moderate (10), moderately severe (15), and severe (20). Moderate exercise was defined as “Do any moderate-intensity sports, fitness, or recreational activities that cause a small increase in breathing or heart rate such as brisk walking, bicycling, swimming, or golf for at least 10 min continuously?”

### 2.5 Statistical analyses

All analyses accounted for the complex survey design of NHANES. Continuous variables were described using mean ± standard deviation (SD), and categorical variables were presented as percentages. The overall cancer prevalence and cancer prevalence across quartiles of dietary creatine intake were calculated. Chi-square tests or Student’s *t*-tests were used to evaluate differences between groups. Multicollinearity diagnostics were performed for all continuous variables, and bidirectional stepwise logistic regression was used to identify independent factors associated with cancer. Additionally, we examined the relationship between age and cancer risk. Multivariable logistic regression analysis was conducted to further analyze the relationship between dietary creatine intake, age, and cancer risk, adjusting for potential confounders including sex, education, family income to poverty ratio, BMI, race/ethnicity, hypertension, drinking status, smoking status, diabetes, PHQ-9, moderate exercise, blood cadmium, and blood lead. Restricted cubic splines (RCS) were employed to visualize the relationships between dietary creatine, age, and cancer risk. Age was incorporated as a covariate in the multivariable logistic regression analysis of dietary creatine intake and cancer risk. Model 1 included no adjustments, Model 2 adjusted for age (median-stratified), sex, education, family income to poverty ratio, BMI, and race/ethnicity, Model 3 additionally adjusted for hypertension, drinking status, smoking status, diabetes, and PHQ-9, and Model 4 included all potential covariates. Models were stratified by sex, age, and BMI to examine the relationship between dietary creatine intake and cancer risk across different populations. Missing values were imputed using sequential mean imputation. Data processing and analysis were performed using IBM SPSS Statistics (Version 24.0) and R (version 4.3.0). A two-sided *p*-value <0.05 was considered statistically significant.

## 3 Results

### 3.1 Cancer prevalence and creatine intake

A total of 25,879 adult participants were included in the analysis, with 12,772 males (49.35%) and 13,107 females (50.65%). The average age was 50.48 years (standard deviation [SD] = 17.65 years). Among the participants, 2,715 individuals were diagnosed with cancer, resulting in an overall cancer prevalence of 10.50 per 100 persons (95% confidence interval [CI]: 10.10–10.90). The average creatine intake for all participants was 0.12 g (SD = 0.09 g). The mean dietary creatine intake for female and male participants was 0.10 g (SD = 0.07 g) and 0.14 g (SD = 0.10 g), respectively. The cancer prevalence among female participants was 10.60 per 100 persons (95% CI: 10.10–11.10), and among male participants, it was 10.40 per 100 persons (95% CI: 9.80–10.90).

**Figure 3:**
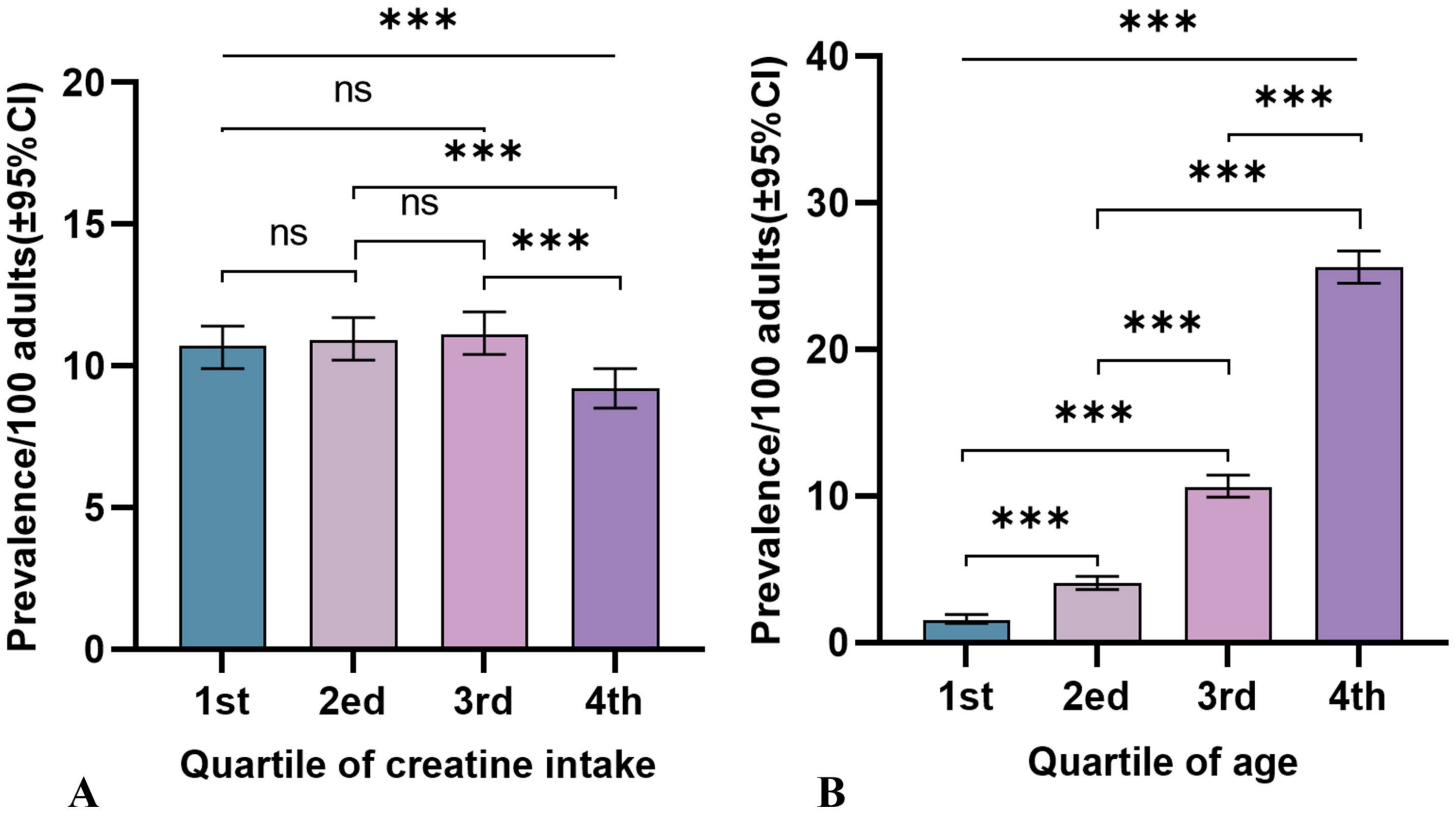
Cancer prevalence rates and 95% confidence intervals among adult participants in NHANES (2007–2018), by quartiles of 2-day average dietary creatine intake and age. **(A)** Dietary creatine intake and cancer prevalence. **(B)** Age and cancer prevalence.

Participants were categorized into quartiles based on their average dietary creatine intake: 0–0.054 g (1st quartile; mean = 0.03 g, SD = 0.01 g), 0.055–0.099 g (2nd quartile; mean = 0.08 g, SD = 0.01 g), 0.100–0.161 g (3rd quartile; mean = 0.13 g, SD = 0.02 g), and 0.162–1.045 g (4th quartile; mean = 0.24 g, SD = 0.09 g). Figure 3 shows the varying cancer prevalence across creatine intake quartiles, with significant differences observed at the highest quartile (Figure 3A). Participants in the lowest quartile had a cancer prevalence of 10.7 per 100 persons (95% CI: 9.90–11.41), while those in the highest quartile had a prevalence of 9.2 per 100 persons (95% CI: 8.50–9.90) (*p* = 0.001).

### 3.2 Participant characteristics by cancer status

Table 1 presents a comparison of the characteristics between participants with and without cancer. A total of 2,715 participants reported having cancer, with an average age of 66.01 years (SD = 13.48 years). Compared to the healthy population, cancer participants were older on average (66.01 vs. 48.66, *p* < 0.001), more likely to be obese (44.97% vs. 22.47%, *p* = 0.010), and had a higher family income to poverty ratio (2.77 vs. 2.51, *p* < 0.001), have hypertension (58.60% vs. 41.40%, *p* < 0.001), be smokers (52.45% vs. 47.55%, *p* < 0.001), be alcohol consumers (28.18% vs. 11.45%, *p* < 0.001), and have higher blood lead levels (average 0.08 vs. 0.07, *p* < 0.001). Significant statistical differences were identified between cancer and non-cancer participants concerning race/ethnicity (*p* < 0.001), age groups (*p* < 0.001), and the quartiles of average dietary creatine intake over two days (*p* = 0.001). No significant differences were found between the groups in terms of sex, PHQ-9 scores, moderate exercise, or blood cadmium levels.

**Table 1:** Characteristics of adult NHANES2007–2018 participants with cancer data (*N* = 25,879).

Characteristic	Overall (*n* = 25,879)	No cancer (*n* = 23,164)	Cancer (*n* = 2,715)	*p*
Age (year)^a^, Mean ± SD	50.48 ± 17.65	48.66 ± 17.18	66.01 ± 13.48	<0.001
Sex^b^, *n*(%)				0.518
Female	13,107 (50.65)	11,716 (50.58)	1,391 (51.23)	
Male	12,772 (49.35)	11,448 (49.42)	1,324 (48.77)	
Age group^b^, *n*(%)				<0.001
20–36 years	6,464 (24.98)	6,362 (27.47)	102 (3.76)	
37–51 years	6,423 (24.82)	6,163 (26.61)	260 (9.58)	
52–65 years	6,486 (25.06)	5,798 (25.03)	688 (25.34)	
66–80 years	6,506 (25.14)	4,841 (20.90)	1,665 (61.33)	
Race/ethnicity^b^, *n*(%)				<0.001
Mexican American	3,522 (13.61)	3,368 (14.54)	154 (5.67)	
Other Hispanic	2,603 (10.06)	2,427 (10.48)	176 (6.48)	
Non-Hispanic and other race	19,754 (76.33)	17,369 (74.98)	2,385 (87.85)	
Education^b^, *n*(%)				<0.001
<9th grade	2,253 (8.71)	2,037 (8.79)	216 (7.96)	
9–11th grade	3,557 (13.74)	3,232 (13.95)	325 (11.97)	
High school diploma/GED	6,055 (23.40)	5,442 (23.49)	613 (22.58)	
Some college/AA degree	7,880 (30.45)	7,047 (30.42)	833 (30.68)	
≥College graduate	6,134 (23.70)	5,406 (23.34)	728 (26.81)	
Family income to poverty ratio^a^, Mean ± SD	2.53 ± 1.55	2.51 ± 1.55	2.77 ± 1.55	<0.001
BMI^b^, *n*(%)				0.010
Normal	5,960 (23.03)	5,350 (23.10)	610 (22.47)	
Underweight	356 (1.38)	312 (1.35)	44 (1.62)	
Overweight	7,361 (28.44)	6,521 (28.15)	840 (30.94)	
Obesity	12,202 (47.15)	10,981 (47.41)	1,221 (44.97)	
PHQ-9^b^, *n*(%)				0.096
Mild	19,803 (76.52)	17,765 (76.69)	2,038 (75.06)	
Moderate	3,862 (14.92)	3,442 (14.86)	420 (15.47)	
Moderately severe	1,351 (5.22)	1,204 (5.20)	147 (5.41)	
Severe	863 (3.33)	753 (3.25)	110 (4.05)	
Hypertension^b^, *n*(%)				<0.001
No	16,091 (62.18)	14,967 (64.61)	1,124 (41.40)	
Yes	9,788 (37.82)	8,197 (35.39)	1,591 (58.60)	
Diabetes^b^, *n*(%)				<0.001
No	21,685 (83.79)	19,642 (84.80)	2,043 (75.25)	
Yes	3,554 (13.73)	2,988 (12.90)	566 (20.85)	
Borderline	640 (2.47)	534 (2.31)	106 (3.90)	
Smoking status^b^, *n*(%)				<0.001
Never smoker	15,188 (58.69)	13,897 (59.99)	1,291 (47.55)	
Current smoker	4,223 (16.32)	3,880 (16.75)	343 (12.63)	
Former smoker	6,468 (24.99)	5,387 (23.26)	1,081 (39.82)	
Drinking status^b^, *n*(%)				<0.001
No	3,180 (12.29)	2,869 (12.39)	311 (11.45)	
Yes	6,368 (24.61)	5,603 (24.19)	765 (28.18)	
NA	16,331 (63.11)	14,692 (63.43)	1,639 (60.37)	
Moderate exercise^b^, *n*(%)				0.155
No	15,390 (59.47)	13,741 (59.32)	1,649 (60.74)	
Yes	10,489 (40.53)	9,423 (40.68)	1,066 (39.26)	
Blood cadmium (nmol/L)^a^, Mean ± SD	4.58 ± 4.61	4.56 ± 4.57	4.73 ± 4.89	0.083
Blood lead (nmol/L)^a^, Mean ± SD	0.07 ± 0.07	0.07 ± 0.07	0.08 ± 0.06	<0.001
Average creatine intake (g)^a^, Mean ± SD	0.12 ± 0.09	0.12 ± 0.09	0.11 ± 0.08	<0.001
Quartile creatine intake^b^, *n*(%)				0.001
1st	6,470 (25.00)	5,779 (24.95)	691 (25.45)	
2nd	6,469 (25.00)	5,762 (24.87)	707 (26.04)	
3rd	6,470 (25.00)	5,749 (24.82)	721 (26.56)	
4th	6,470 (25.00)	5,874 (25.36)	596 (21.95)	

a Student *t*-test.

b Chi-square test.

### 3.3 Relationship between age and cancer prevalence

**Figure 4:**
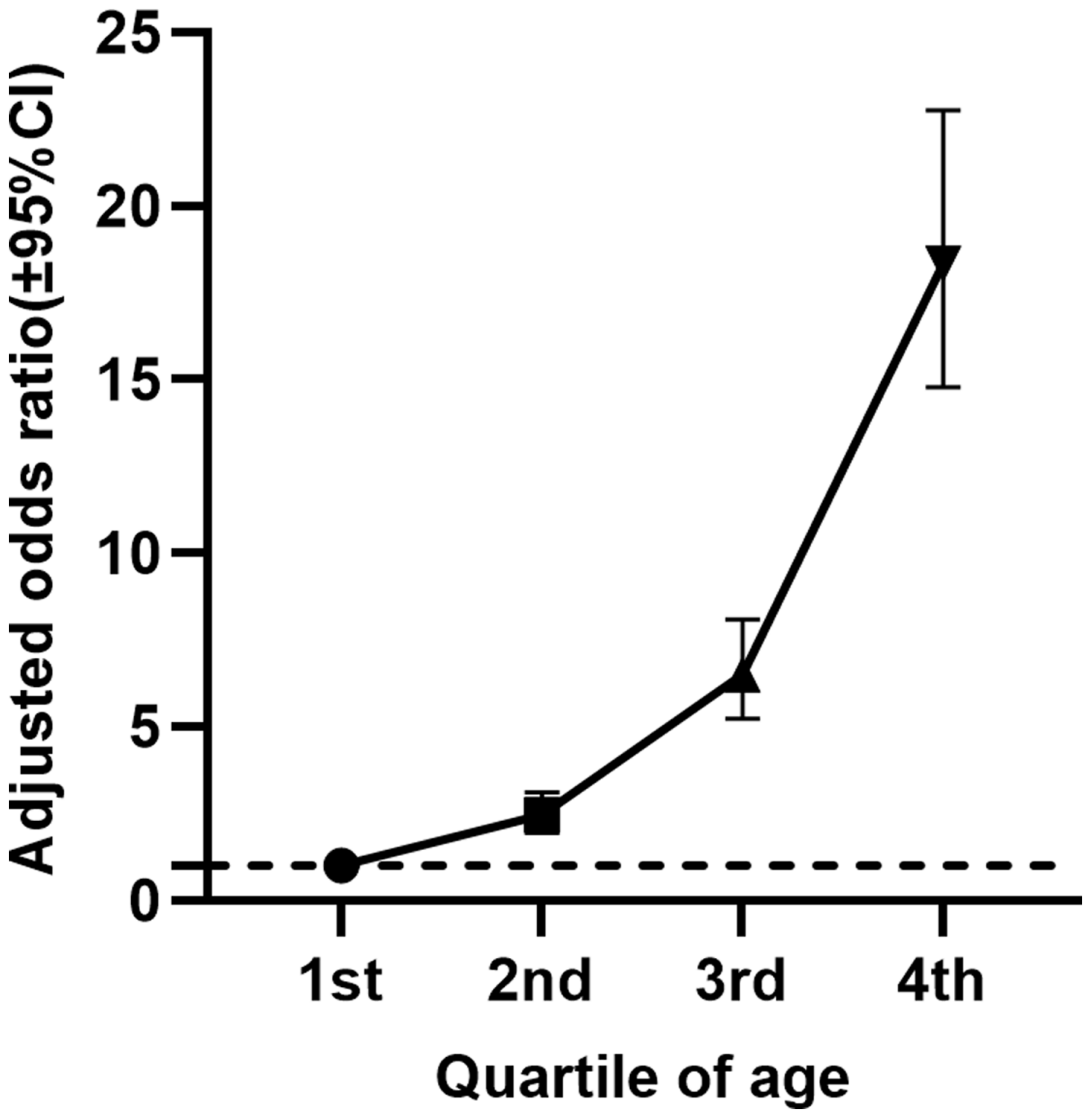
Relationship between age quartiles of NHANES participants (2007–2018) and cancer risk (adjusted odds ratio and 95% confidence interval).

Table 2 reports the cancer incidence associated with age, age quartiles, and 2-day average dietary creatine intake, without considering their potential interaction (Figure 3B). The crude model shows a positive relationship between age and cancer prevalence (OR = 1.07, 95% CI: 1.07–1.07, *p* < 0.001), and this relationship remains significant after standardizing age (OR = 3.31, 95% CI: 3.14–3.50, *p* < 0.001). Model 4, which adjusts for potential confounders including sex, education, family income to poverty ratio, BMI, race/ethnicity, hypertension, drinking status, smoking status, diabetes, PHQ-9, moderate exercise, blood cadmium, and blood lead, still shows a stable positive relationship between age and cancer risk (adjusted OR = 1.07, 95% CI: 1.07–1.07, *p* < 0.001). The standardized age model reports a 3.27-fold increase in cancer risk for each standard deviation increase in age (SD = 17.65) (adjusted OR = 3.27, 95% CI: 3.07–3.48, *p* < 0.001). Grouping age into quartiles further validates our hypothesis. Particularly, participants aged 66–80 years had an 18.34-fold higher cancer risk compared to those aged 20–36 years (adjusted OR = 18.34, 95% CI: 14.78–22.76, *p* < 0.001). Figure 4 shows the relationship between different age groups and cancer risk.

**Table 2:** Independent associations between 2-day average dietary creatine intake and cancer risk by age in adult participants of NHANES (2007–2018).

Variables	Model1		Model2		Model3		Model4	
	OR (95%CI)	*p*	OR (95%CI)	*p*	OR (95%CI)	*p*	OR (95%CI)	*p*
Average creatine intake, continuous	0.36 (0.23 ~ 0.57)	<0.001	0.32 (0.20 ~ 0.52)	<0.001	0.36 (0.22 ~ 0.59)	<0.001	0.36 (0.22 ~ 0.60)	<0.001
Per SD increase	0.91 (0.87 ~ 0.95)	<0.001	0.90 (0.86 ~ 0.94)	<0.001	0.91 (0.87 ~ 0.95)	<0.001	0.91 (0.87 ~ 0.95)	<0.001
Age, continuous	1.07 (1.07 ~ 1.07)	<0.001	1.07 (1.07 ~ 1.07)	<0.001	1.07 (1.07 ~ 1.07)	<0.001	1.07 (1.07 ~ 1.07)	<0.001
Per SD increase	3.31 (3.14 ~ 3.50)	<0.001	3.33 (3.15 ~ 3.52)	<0.001	3.24 (3.05 ~ 3.44)	<0.001	3.27 (3.07 ~ 3.48)	<0.001
Age group								
20–36 years	1.00 (Reference)		1.00 (Reference)		1.00 (Reference)		1.00 (Reference)	
37–51 years	2.63 (2.09 ~ 3.32)	<0.001	2.61 (2.07 ~ 3.30)	<0.001	2.45 (1.94 ~ 3.10)	<0.001	2.46 (1.95 ~ 3.11)	<0.001
52–65 years	7.40 (5.99 ~ 9.14)	<0.001	7.49 (6.06 ~ 9.27)	<0.001	6.48 (5.22 ~ 8.05)	<0.001	6.52 (5.24 ~ 8.10)	<0.001
66–80 years	21.45 (17.51 ~ 26.29)	<0.001	21.59 (17.58 ~ 26.51)	<0.001	18.23 (14.72 ~ 22.57)	<0.001	18.34 (14.78 ~ 22.76)	<0.001

OR, Odds Ratio; CI, Confidence Interval.

Model1: Crude.

Model2: Adjust: Sex, Education, Family income to poverty ratio, BMI, Race/ethnicity.

Model3: Adjust: Sex, Education, Family income to poverty ratio, BMI, Race/ethnicity, Hypertension, Drinking status, Smoking status, Diabetes, PHQ-9.

Model4: Adjust: Sex, Education, Family income to poverty ratio, BMI, Race/ethnicity, Hypertension, Drinking status, Smoking status, Diabetes, PHQ-9, Moderate exercise, Blood cadmium, Blood lead.

Additionally, in the absence of age consideration, the crude model indicates a protective effect of average dietary creatine against cancer (OR = 0.36, 95% CI: 0.23–0.57, *p* < 0.001). After adjusting for potential confounders, the standardized creatine intake remains a protective factor against cancer (adjusted OR = 0.91, 95% CI: 0.87–0.95, *p* < 0.001).

### 3.4 Linear and nonlinear relationships between age, dietary creatine intake and cancer risk

**Figure 5:**
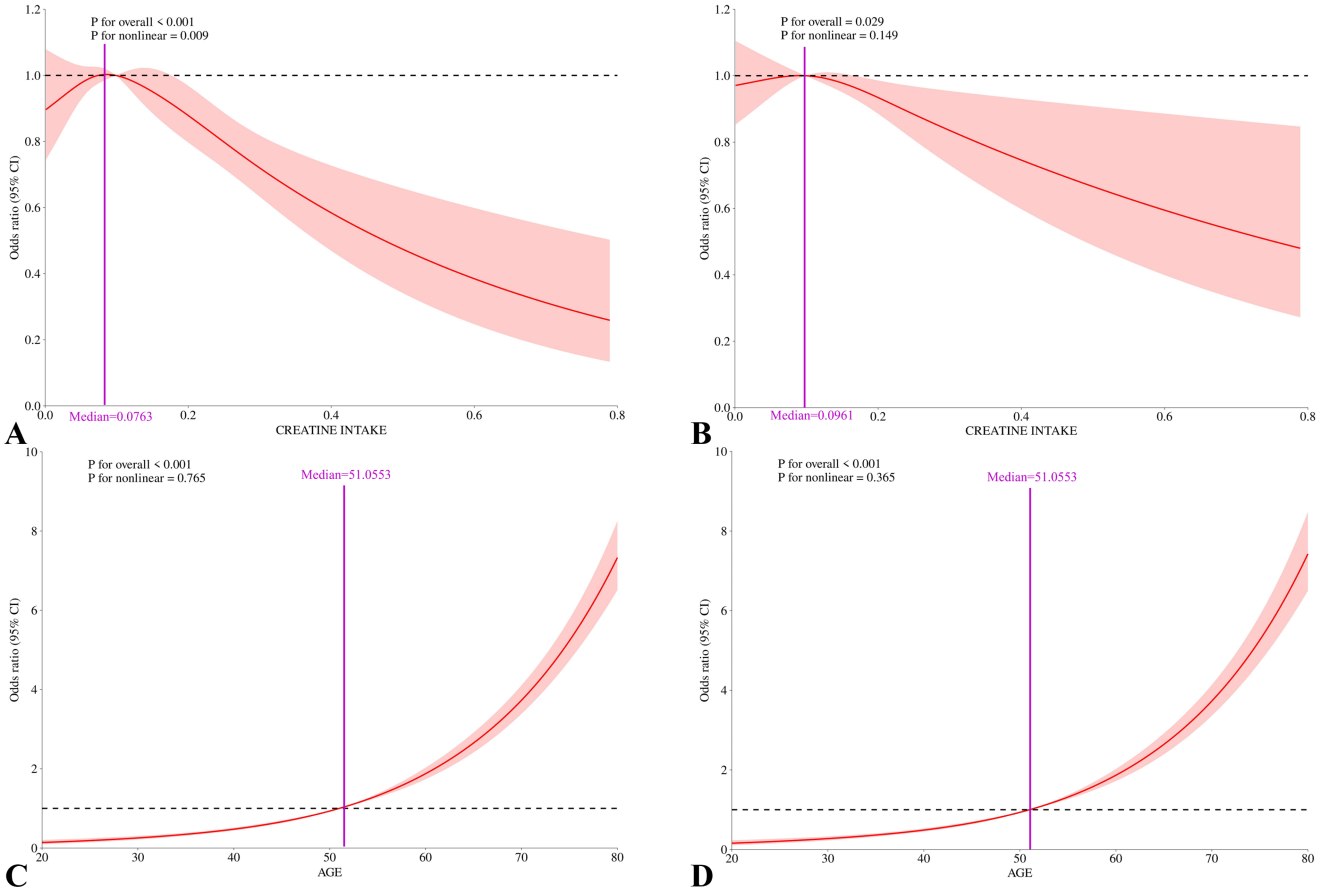
Nonlinear associations between 2-day average dietary creatine intake and age with cancer risk among adult participants in NHANES (2007–2018). **(A)** Restricted cubic splines for the association between 2-day average dietary creatine intake and cancer risk, crude model. **(B)** Restricted cubic splines for the association between 2-day average dietary creatine intake and cancer risk, adjusted for covariates. **(C)** Restricted cubic splines for the association between age and cancer risk, crude model. **(D)** Restricted cubic splines for the association between age and cancer risk, adjusted for covariates.

We conducted RCS analysis to better illustrate the relationships between age, dietary creatine intake, and cancer. The RCS analysis results show a J-shaped association between age and cancer risk (*p* for nonlinear = 0.765 and *p* for overall <0.001), and a non-linear relationship between average dietary creatine intake and cancer risk, characterized by an initial increase followed by a decrease (*p* for nonlinear = 0.009 and *p* for overall <0.001), indicating that cancer prevalence initially increases with creatine intake and then decreases. After adjusting for potential confounding factors, the nonlinear relationship between two-day dietary creatine intake and cancer risk was no longer significant (*p* for nonlinear = 0.149, *p* for overall = 0.029). In contrast, the relationship between age and cancer risk remained stable (*p* for nonlinear = 0.365, *p* for overall <0.001). (Figure 5).

### 3.5 Relationship between creatine and cancer

Based on the RCS analysis results, the median age of 51.0553 was used to categorize participants into age groups for further analysis. As shown in Table 3, in the crude model, the average 2-day dietary creatine intake had a protective effect against cancer (OR = 0.36, 95% CI: 0.23–0.57, *p* < 0.001). After adjusting for confounders, including age, the association between creatine intake and cancer incidence remained significant (adjusted OR = 0.95, 95% CI: 0.91–0.99, *p* = 0.025). Specifically, each standard deviation increase in dietary creatine intake was associated with a 5% reduction in cancer risk.

**Table 3:** Joint associations between 2-day average dietary creatine intake and age with cancer risk in adult participants of NHANES (2007–2018).

Variables	Model1		Model2		Model3		Model4		*p* for interaction
	OR (95%CI)	*p*	OR (95%CI)	*p*	OR (95%CI)	*p*	OR (95%CI)	*p*	
Average creatine intake, continuous	0.36 (0.23 ~ 0.57)	<0.001	0.58 (0.35 ~ 0.95)	0.032	0.56 (0.34 ~ 0.93)	0.025	0.56 (0.34 ~ 0.93)	0.025	
Per SD increase	0.91 (0.87 ~ 0.95)	<0.001	0.95 (0.91 ~ 0.99)	0.032	0.95 (0.90 ~ 0.99)	0.025	0.95 (0.91 ~ 0.99)	0.025	
Quartile creatine intake									
1st	1.00 (Reference)		1.00 (Reference)		1.00 (Reference)		1.00 (Reference)		
2nd	1.03 (0.92 ~ 1.15)	0.648	1.01 (0.90 ~ 1.14)	0.822	1.02(0.90 ~ 1.14)	0.807	1.00 (0.90 ~ 1.13)	0.873	
3rd	1.05 (0.94 ~ 1.17)	0.398	1.03 (0.92 ~ 1.15)	0.645	1.02 (0.91 ~ 1.15)	0.758	1.00 (0.89 ~ 1.12)	0.993	
4th	0.85 (0.76 ~ 0.95)	0.005	0.93 (0.82 ~ 1.05)	0.249	0.92 (0.81 ~ 1.04)	0.186	0.84 (0.75 ~ 0.95)	0.005	
Age-group stratified models									0.005
20–51 years^a^									
Per SD increase	0.84 (0.76 ~ 0.94)	0.002	0.95 (0.85 ~ 1.07)	0.416	0.96 (0.86 ~ 1.08)	0.510	0.96 (0.86 ~ 1.08)	0.508	
52–80 years^a^									
Per SD increase	0.99 (0.94 ~ 1.04)	0.570	0.88 (0.79 ~ 0.99)	0.032	0.89 (0.80 ~ 1.00)	0.052	0.89 (0.80 ~ 1.00)	0.051	
Sex stratified models									0.191
Females^b^									
Per SD increase	0.96 (0.89 ~ 1.03)	0.231	0.99 (0.92 ~ 1.07)	0.851	0.99 (0.92 ~ 1.07)	0.810	0.99 (0.92 ~ 1.07)	0.817	
Males^b^									
Per SD increase	0.88 (0.84 ~ 0.93)	<0.001	0.93 (0.88 ~ 0.99)	0.020	0.93 (0.88 ~ 0.99)	0.021	0.93 (0.88 ~ 0.99)	0.021	
BMI-group stratified models									<0.001
Normal^c^									
Per SD increase	0.95 (0.87 ~ 1.04)	0.257	1.01 (0.92 ~ 1.12)	0.832	1.00 (0.91 ~ 1.11)	0.924	1.01 (0.91 ~ 1.12)	0.854	
Underweight^c^									
Per SD increase	1.34 (0.93 ~ 1.94)	0.120	1.52 (0.99 ~ 2.32)	0.053	1.60 (1.02 ~ 2.50)	0.039	1.81(1.13 ~ 2.92)	0.014	
Overweight^c^									
Per SD increase	0.85 (0.79 ~ 0.92)	<0.001	0.91 (0.84 ~ 0.99)	0.035	0.92 (0.84 ~ 0.99)	0.045	0.92 (0.84 ~ 0.99)	0.044	
Obesity^c^									
Per SD increase	0.92 (0.87 ~ 0.98)	0.013	0.94 (0.88 ~ 1.00)	0.067	0.93 (0.87 ~ 0.99)	0.043	0.94 (0.87 ~ 1.00)	0.052	

OR, Odds Ratio; CI, Confidence Interval.

Model1: Crude.

Model2: Adjust: Age(Stratified by median), Sex, Education, Family income to poverty ratio, BMI, Race/ethnicity.

Model3: Adjust: Age(Stratified by median), Sex, Education, Family income to poverty ratio, BMI, Race/ethnicity, Hypertension, Drinking status, Smoking status, Diabetes, PHQ-9.

Model4: Adjust: Age(Stratified by median), Sex, Education, Family income to poverty ratio, BMI, Race/ethnicity, Hypertension, Drinking status, Smoking status, Diabetes, PHQ-9, Moderate exercise, Blood cadmium, Blood lead.

a Adjusted models do not include age.

b Adjusted models do not include sex.

c Adjusted models do not include BMI.

In the age-stratified model, this protective effect was not statistically significant for participants aged 20–51 years (adjusted OR = 0.96, 95% CI: 0.86–1.08, *p* = 0.508) or those aged 52–80 years (adjusted OR = 0.89, 95% CI: 0.80–1.00, *p* = 0.051). In sex-stratified models, the adjusted odds ratio for females was also not statistically significant (adjusted OR = 0.99, 95% CI: 0.92–1.07, *p* = 0.817). However, a significant protective association was observed between dietary creatine intake and cancer risk in males. For each standard deviation increase in creatine intake, cancer risk decreased by approximately 7% in male participants (adjusted OR = 0.93, 95% CI: 0.88–0.99, *p* = 0.021). Similarly, a significant inverse correlation was found between dietary creatine intake and cancer risk in overweight individuals. Each standard deviation increase in creatine intake was linked to an 8% decrease in cancer risk (adjusted OR = 0.92, 95% CI: 0.84–0.99, *p* = 0.044). In contrast, underweight individuals showed an opposite trend, with higher dietary creatine intake being associated with increased cancer risk (adjusted OR = 1.81, 95% CI: 1.13–2.92, *p* = 0.014).

**Figure 6:**
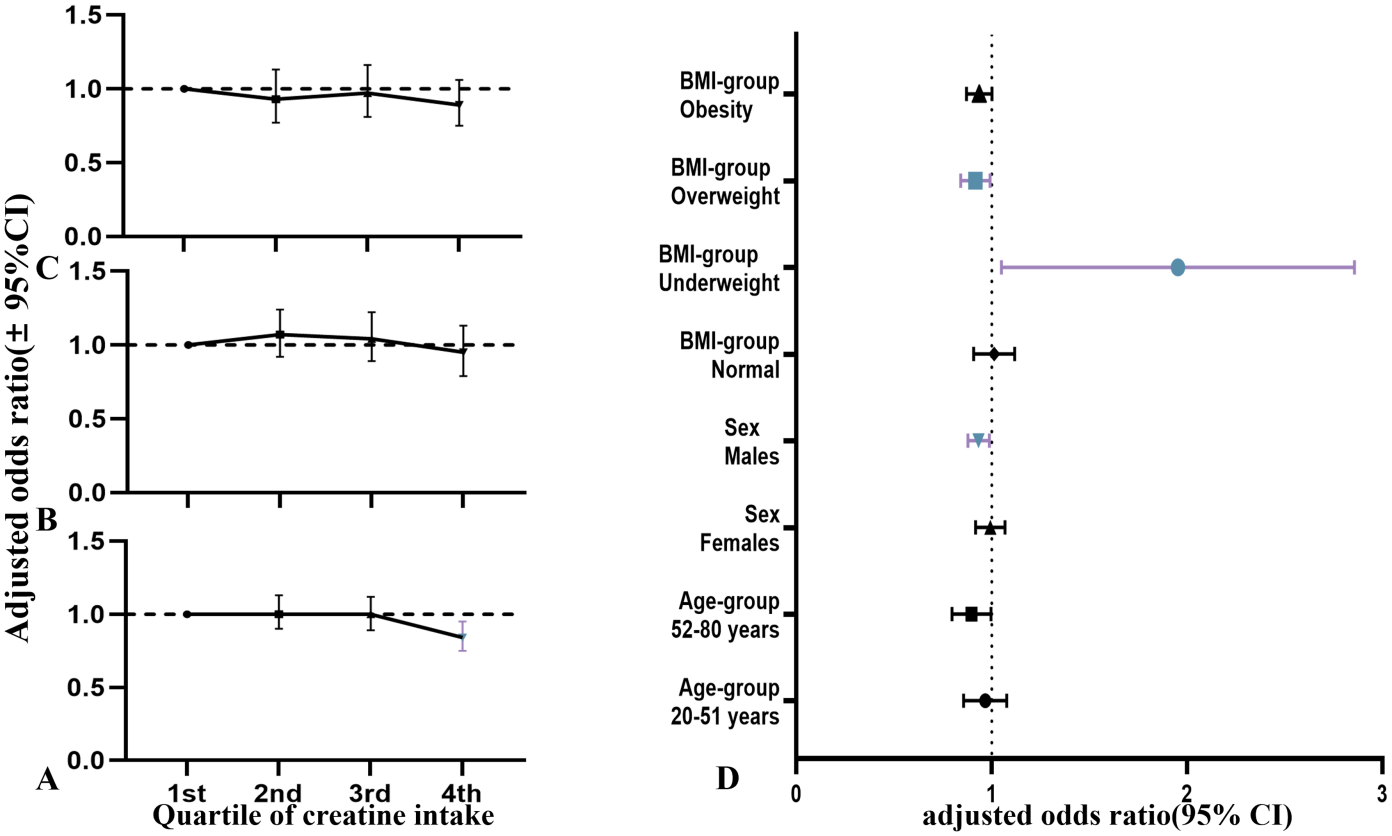
Relationship between quartiles of dietary creatine intake and cancer risk among NHANES participants (2007–2018), stratified by gender (adjusted odds ratios and 95% confidence intervals). **(A)** Dietary creatine intake and cancer risk in all participants. **(B)** Dietary creatine intake and cancer risk in female participants. **(C)** Dietary creatine intake and cancer risk in male participants. **(D)** Forest plot of age, gender, and BMI subgroups.

In the overall study population, participants in the fourth quartile of dietary creatine intake had a lower risk of cancer compared to those in the first quartile (adjusted OR = 0.84, 95% CI: 0.75–0.95, *p* = 0.005). Figure 6 illustrates the relationship between the quartiles of average 2-day dietary creatine intake and cancer risk, though this association was not statistically significant when participants were stratified by age, sex, or BMI.

**Figure 7:**
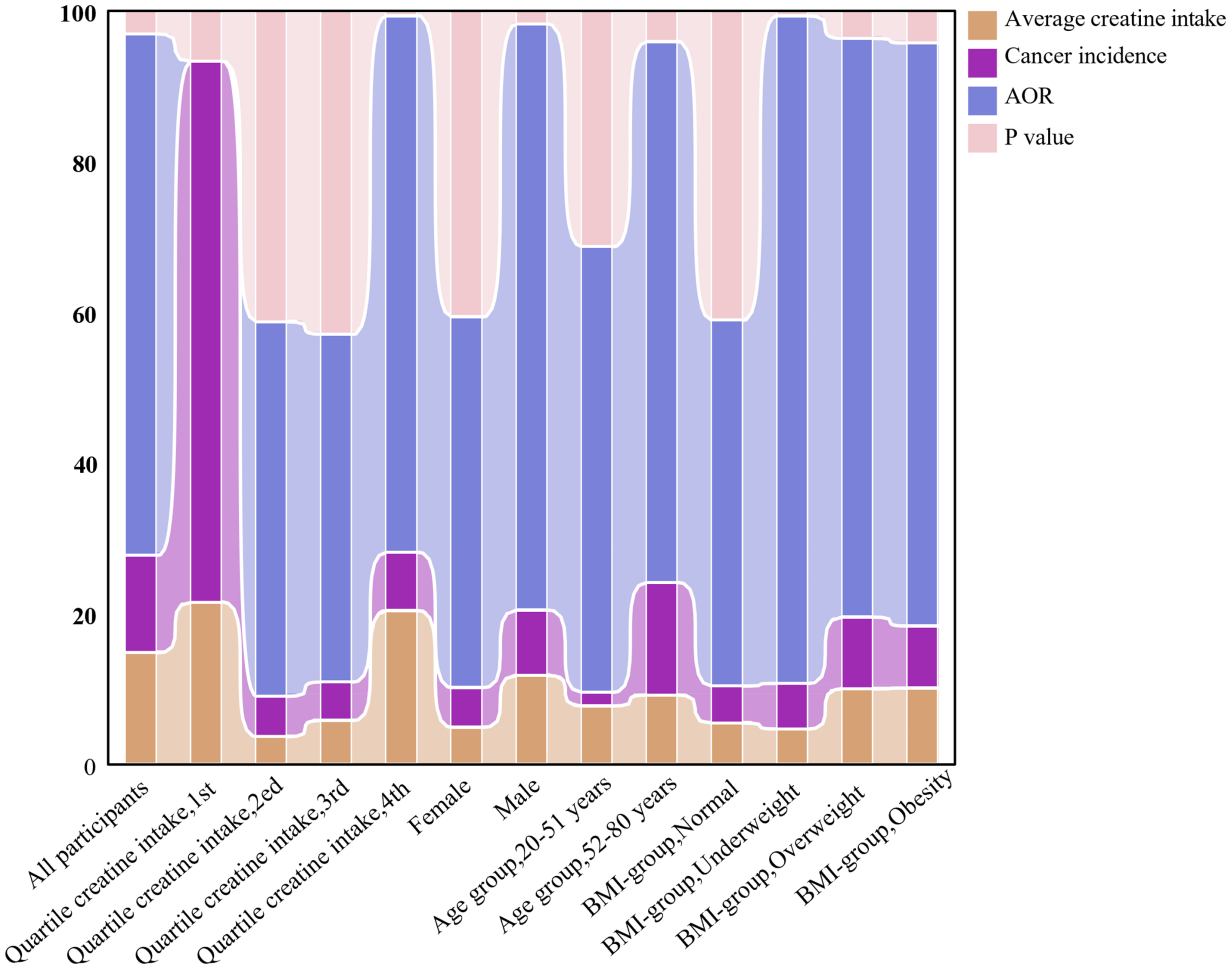
The evolving relationship between dietary creatine intake and cancer risk among various groups of NHANES participants (2007–2018).

The interaction analysis revealed a significant interaction effect between dietary creatine intake, BMI, and age (*p* < 0.001, *p* = 0.005). Further analysis suggested that higher dietary creatine intake correlated with reduced cancer risk, particularly in the elderly population (Table 4). Figure 7 presents the average dietary creatine intake, cancer prevalence, odds ratio (OR), and *p*-value across different groups as percentages.

**Table 4:** Two day average dietary creatine intake and its association with cancer risk across various age groups.

Variables	Crude model		Adjusted model	
	OR (95%CI)	*p*	OR (95%CI)	*p*
Age-group stratified models				
20–51 years average creatine intake				
1st				
Per SD increase	1.32 (0.39 ~ 4.46)	0.657	1.41 (0.40 ~ 4.93)	0.594
2nd				
Per SD increase	0.63 (0.17 ~ 2.34)	0.490	0.77 (0.20 ~ 2.92)	0.698
3rd				
Per SD increase	0.97 (0.35 ~ 2.67)	0.958	1.13(0.41 ~ 3.14)	0.818
4th				
Per SD increase	0.89 (0.69 ~ 1.14)	0.351	1.01 (0.78 ~ 1.30)	0.964
52–80 years average creatine intake				
1st				
Per SD increase	1.49 (0.79 ~ 2.84)	0.219	1.32 (0.68 ~ 2.55)	0.407
2nd				
Per SD increase	0.89 (0.45 ~ 1.75)	0.732	0.72 (0.36 ~ 1.45)	0.359
3rd				
Per SD increase	0.87 (0.53 ~ 1.40)	0.558	0.87 (0.53 ~ 1.43)	0.573
4th				
Per SD increase	0.87 (0.78 ~ 0.98)	0.017	0.86 (0.77 ~ 0.97)	0.014

OR, Odds Ratio; CI, Confidence Interval.

Crude model: Crude.

Adjusted model: Sex, Education, Family income to poverty ratio, BMI, Race/ethnicity, Hypertension, Drinking status, Smoking status, Diabetes, PHQ-9, Moderate exercise, Blood cadmium, Blood lead.

As shown in Table 4, dietary creatine intake was further subdivided into detailed groups within different age brackets. Among elderly participants in the fourth quartile of 2-day dietary creatine intake, cancer risk decreased by 13% for every standard deviation increase in creatine intake (OR = 0.87, 95% CI: 0.78–0.98, *p* = 0.017). After adjusting for potential confounders, the model remained robust, with a 14% reduction in cancer risk for every standard deviation increase in creatine intake (adjusted OR = 0.86, 95% CI: 0.77–0.97, *p* = 0.014).

## 4 Discussion

To our knowledge, this is the first study using a nationally representative sample from the United States to investigate the relationship between age, dietary creatine intake, and cancer risk. Our findings revealed a significant negative correlation between dietary creatine intake and cancer risk (Figure 5). After controlling for potential confounders, each one standard deviation increase in dietary creatine intake was associated with an approximately 5% decrease in cancer risk. This association remained significant across various levels of dietary creatine intake (Figures 3, 6). Notably, among participants aged 52 and above in the highest quartile of dietary creatine intake, each additional 0.09 g of creatine was linked to a 14% reduction in cancer risk (Table 4).

Creatine, a nitrogen-containing organic acid synthesized in the liver from L-arginine, can also be obtained through dietary sources (7, 16, 18). Cancer development is associated with mechanisms such as immune evasion, oxidative stress, and chronic inflammation—processes in which creatine plays a beneficial role. Specifically, creatine inhibits the interaction between the IFN-*γ* receptor and JAK2, thereby blocking JAK-STAT1 signaling and suppressing the expression of pro-inflammatory genes (17). Additionally, creatine reduces the release of various inflammatory factors, such as TNF-*α* and IL-1β, and decreases the generation of reactive oxygen species (ROS), exerting anti-inflammatory effects (13, 15). Furthermore, creatine modulates immune function by promoting the generation of regulatory T cells (Treg) and Th2 cells, inhibiting excessive macrophage activation, and enhancing the secretion of anti-inflammatory cytokines (4, 5, 12, 15). These findings align with our results and provide a basis for understanding the potential therapeutic value of creatine.

Our study also identified gender differences in the relationship between dietary creatine intake and cancer risk. While the average 2-day dietary creatine intake was closely associated with reduced cancer risk in the overall population, this relationship varied by gender. An inverse relationship between creatine intake and cancer risk was particularly evident among male participants. For every 0.09 g increase in creatine intake, the cancer incidence in males decreased by 7%, compared to a 2.2% reduction across all participants. Previous studies have also shown that phosphocreatine concentrations are lower in healthy women than in healthy men (9, 11). Animal studies have demonstrated that creatine treatment slows tumor growth and reduces liver cell damage in Wistar Walker 256 tumor-bearing rats (21, 22). However, since these studies were conducted exclusively on male rats, caution is required when interpreting gender differences in creatine’s effects on tumor growth.

Hormones play a significant role in driving human behavior. After puberty, testosterone levels in males increase approximately 30-fold, compared to a 15-fold increase in females (9). Skeletal muscle serves as a storage site for phosphocreatine, and the body’s phosphocreatine content is influenced by skeletal muscle mass. Evidence suggests that androgens like testosterone stimulate the proliferation and differentiation of myocytes in mouse skeletal muscle via the androgen receptor (AR) signaling pathway (23, 24). Hormonal differences between genders may explain the variation in creatine levels between males and females. However, the observed gender differences in cancer prevalence contradict our initial hypotheses. Notably, cancer incidence is about 2.3% higher in males than females (212.67/100,000 vs. 208.08/100,000), and male cancer mortality is significantly higher than female mortality (127.70/100,000 vs. 68.67/100,000). The National Cancer Institute in the United States found that men have a 20% higher risk of cancer than women (25).

Evidence suggests that male hormones contribute to this gender disparity in cancer risk (24, 25). Male hormones activate the AR signaling pathway, which regulates the Tcf7 transcription factor, leading to dysfunction in stem cell-like CD8+ T cells, excluding reproductive system tumors. Further analysis indicates that the source of these hormonal differences may be related to sex chromosomes. Research has identified EXITS (Escape from X-inactivation Tumor Suppressor) “insurance genes” in females that reduce cancer risk (26). The role of androgens in this context remains ambiguous, warranting further research to clarify the relationship between androgens, creatine, and their impact on cancer.

Preliminary evidence also suggests that adipocytes may influence this relationship, particularly in underweight or overweight individuals. Moderate levels of adipose tissue may offer protective benefits in the link between dietary creatine intake and cancer risk. Obesity is regarded as a chronic low-grade inflammatory state, with adipocytes secreting adipokines and inflammatory factors like IL-6 and TNF-*α*, which promote cellular carcinogenesis (27). Excess adipocytes can also impair immune function by inhibiting CD8+ T cells (28). Studies showed that adipose tissue near breast cancer secreted IGFBP2 (Insulin-like Growth Factor Binding Protein 2), which affected IGF-II autocrine signaling in cancer cells, thereby inhibiting the progression of breast cancer (29). In contrast, underweight individuals exhibit a positive correlation between dietary creatine intake and cancer risk. This may be attributed to factors like malnutrition, chronic disease, and weakened immune function, which have a more substantial impact on cancer risk than creatine intake alone. These findings underscore the complex role of adipose tissue in cancer risk.

The study also found a significant positive correlation between age and cancer risk. Individuals over the age of 66 had a cancer incidence rate 18.34 times higher than those aged 20–36, peaking at 85 years old (30). This trend aligns with our findings. Aging is an irreversible process marked by genomic instability, epigenetic alterations, chronic inflammation, and metabolic dysregulation, all of which contribute to cancer development and represent the “meta-hallmarks” of aging and cancer (6). Although aging is inevitable, cancer prevention can still be pursued through lifestyle changes such as quitting smoking and reducing alcohol consumption.

Despite these findings, the study acknowledges several limitations. First, there are concerns regarding the validity of dietary intake data from the NHANES database. Dietary patterns, nutrient absorption, and metabolism may differ between cancer patients and healthy individuals. The American Cancer Society recommends that cancer survivors reduce their red meat consumption during and after treatment (31). Since dietary creatine in this study was derived primarily from various meat proteins, cancer participants may have lower dietary creatine intake due to this recommendation. Additionally, the exclusion of non-meat protein sources could lead to discrepancies between reported and actual creatine intake. Furthermore, the study did not examine the role of other nutrients, such as vitamins and tryptophan, found in meat proteins and their potential influence on cancer risk. For instance, vitamin D and omega-3 fatty acids, commonly found in fish and seafood, have been linked to cancer prevention. The use of external creatine supplements was also not considered. Although the population using these supplements is small and primarily consists of regular athletes, this factor may have influenced the results, potentially skewing them towards protective effects. This study utilized the mean imputation method to handle missing values, which effectively increased the sample size. However, when data are not missing at random, mean imputation can introduce bias into the results. This bias may obscure relationships between variables, ultimately affecting the study’s outcomes (32). Future research should employ more advanced imputation techniques, such as Bayesian multiple imputation, to minimize potential bias.

The strength of this study lies in its use of nationally representative NHANES data and the inclusion of several cancer risk factors, such as depression, unhealthy lifestyles (e.g., smoking, drinking), occupational or environmental exposure (e.g., cadmium, lead), hypertension, and diabetes. Nonetheless, although the results demonstrate a negative correlation between dietary creatine intake and cancer risk, correlation does not imply causation. Unmeasured variables or confounding factors may have influenced the observed relationship. Rigorous clinical trials or long-term observational studies are required to determine whether dietary creatine intake genuinely reduces cancer risk.

## 5 Conclusion

Our study identified a significant linear negative correlation between dietary creatine intake and cancer risk among U.S. adults, particularly in males and overweight individuals. Age remains a key factor influencing cancer risk. Future research should explore the potential therapeutic value of dietary creatine, providing new insights into cancer prevention and treatment.

## Data Availability

The datasets presented in this study can be found in online repositories. The names of the repository/repositories and accession number(s) can be found at: https://wwwn.cdc.gov/nchs/nhanes/Default.aspx.

## References

[1] Dizon DS Kamal AH. Cancer statistics 2024: all hands on deck. CA Cancer J Clin. (2024) 74:8–9. doi: 10.3322/caac.21824, PMID: 38230825

[2] GBD 2019 Cancer Risk Factors Collaborators. The global burden of cancer attributable to risk factors, 2010-19: a systematic analysis for the global burden of disease study 2019. Lancet. (2022) 400:563–91. doi: 10.1016/S0140-6736(22)01438-6, PMID: 35988567 PMC9395583

[3] Loo JM Scherl A Nguyen A Man FY Weinberg E Zeng Z. Extracellular metabolic energetics can promote cancer progression. Cell. (2015) 160:393–406. doi: 10.1016/j.cell.2014.12.018, PMID: 25601461 PMC4312495

[4] Di Biase S Ma X Wang X Yu J Wang YC Smith DJ. Creatine uptake regulates CD8 T cell antitumor immunity. J Exp Med. (2019) 216:2869–82. doi: 10.1084/jem.20182044, PMID: 31628186 PMC6888972

[5] Li B Yang L. Creatine in T cell antitumor immunity and Cancer immunotherapy. Nutrients. (2021) 13:1633. doi: 10.3390/nu13051633, PMID: 34067957 PMC8152274

[6] López-Otín C Pietrocola F Roiz-Valle D Galluzzi L Kroemer G. Meta-hallmarks of aging and cancer. Cell Metab. (2023) 35:12–35. doi: 10.1016/j.cmet.2022.11.001, PMID: 36599298

[7] Zhang L Bu P. The two sides of creatine in cancer. Trends Cell Biol. (2022) 32:380–90. doi: 10.1016/j.tcb.2021.11.004, PMID: 34895811

[8] Tarnopolsky MA MacLennan DP. Creatine monohydrate supplementation enhances high-intensity exercise performance in males and females. Int J Sport Nutr Exerc Metab. (2000) 10:452–63. doi: 10.1123/ijsnem.10.4.452, PMID: 11099372

[9] Hunter SK Angadi SS Bhargava A Harper J Hirschberg AL Levine BD. The biological basis of sex differences in athletic performance: consensus statement for the American College of Sports Medicine. Med Sci Sports Exerc. (2023) 55:2328–60. doi: 10.1249/MSS.0000000000003300, PMID: 37772882

[10] Bender A Koch W Elstner M Schombacher Y Bender J Moeschl M. Creatine supplementation in Parkinson disease: a placebo-controlled randomized pilot trial. Neurology. (2006) 67:1262–4. doi: 10.1212/01.wnl.0000238518.34389.12, PMID: 17030762

[11] Bakian AV Huber RS Scholl L Renshaw PF Kondo D. Dietary creatine intake and depression risk among U.S. adults. Transl Psychiatry. (2020) 10:52. doi: 10.1038/s41398-020-0741-x, PMID: 32066709 PMC7026167

[12] Peng Z Saito S. Creatine supplementation enhances anti-tumor immunity by promoting adenosine triphosphate production in macrophages. Front Immunol. (2023) 14:1176956. doi: 10.3389/fimmu.2023.1176956, PMID: 37662917 PMC10471797

[13] Wei L Wang R Lin K Jin X Li L Wazir J. Creatine modulates cellular energy metabolism and protects against cancer cachexia-associated muscle wasting. Front Pharmacol. (2022) 13:1086662. doi: 10.3389/fphar.2022.1086662, PMID: 36569317 PMC9767983

[14] Kurth I Yamaguchi N Andreu-Agullo C Tian HS Sridhar S Takeda S. Therapeutic targeting of SLC6A8 creatine transporter suppresses colon cancer progression and modulates human creatine levels. Sci Adv. (2021) 7:eabi7511. doi: 10.1126/sciadv.abi7511 34613776 PMC8494442

[15] Bredahl EC Eckerson JM Tracy SM McDonald TL Drescher KM. The role of creatine in the development and activation of immune responses. Nutrients. (2021) 13:751. doi: 10.3390/nu13030751, PMID: 33652752 PMC7996722

[16] Kazak L Cohen P. Creatine metabolism: energy homeostasis, immunity and cancer biology. Nat Rev Endocrinol. (2020) 16:421–36. doi: 10.1038/s41574-020-0365-5, PMID: 32493980

[17] Campos-Ferraz PL Gualano B das Neves W Andrade IT Hangai I RTS P. Exploratory studies of the potential anti-cancer effects of creatine. Amino Acids. (2016) 48:1993–2001. doi: 10.1007/s00726-016-2180-9, PMID: 26872655

[18] Brosnan ME Brosnan JT. The role of dietary creatine. Amino Acids. (2016) 48:1785–91. doi: 10.1007/s00726-016-2188-1 26874700

[19] Zhang L Zhu Z Yan H Wang W Wu Z Zhang F. Creatine promotes cancer metastasis through activation of Smad2/3. Cell Metab. (2021) 33:1111–1123.e4. doi: 10.1016/j.cmet.2021.03.009, PMID: 33811821

[20] Ostojic SM Grasaas E Cvejic J. Dietary creatine and cancer risk in the U.S. population: NHANES 2017–2020. J Funct Foods. (2023) 108:105733. doi: 10.1016/j.jff.2023.105733, PMID: 39726436

[21] Cella PS Marinello PC Borges FH Ribeiro DF Chimin P Testa MTJ. Creatine supplementation in Walker-256 tumor-bearing rats prevents skeletal muscle atrophy by attenuating systemic inflammation and protein degradation signaling. Eur J Nutr. (2020) 59:661–9. doi: 10.1007/s00394-019-01933-6, PMID: 30806774

[22] Deminice R Cella PS Padilha CS Borges FH da Silva LE Campos-Ferraz PL. Creatine supplementation prevents hyperhomocysteinemia, oxidative stress and cancer-induced cachexia progression in Walker-256 tumor-bearing rats. Amino Acids. (2016) 48:2015–24. doi: 10.1007/s00726-016-2172-9, PMID: 26781304

[23] Spiering BA Kraemer WJ Vingren JL Ratamess NA Anderson JM Armstrong LE. Elevated endogenous testosterone concentrations potentiate muscle androgen receptor responses to resistance exercise. J Steroid Biochem Mol Biol. (2009) 114:195–9. doi: 10.1016/j.jsbmb.2009.02.005, PMID: 19429451

[24] Yang C Jin J Yang Y Sun H Wu L Shen M. Androgen receptor-mediated CD8(+) T cell stemness programs drive sex differences in antitumor immunity. Immunity. (2022) 55:1747. doi: 10.1016/j.immuni.2022.07.016, PMID: 36103859

[25] Kwon H Schafer JM Song NJ Kaneko S Li A Xiao T. Androgen conspires with the CD8(+) T cell exhaustion program and contributes to sex bias in cancer. Sci Immunol. (2022) 7:eabq2630. doi: 10.1126/sciimmunol.abq2630, PMID: 35420889 PMC9374385

[26] Dunford A Weinstock DM Savova V Schumacher SE Cleary JP Yoda A. Tumor-suppressor genes that escape from X-inactivation contribute to cancer sex bias. Nat Genet. (2017) 49:10–6. doi: 10.1038/ng.3726, PMID: 27869828 PMC5206905

[27] Cypess AM. Reassessing human adipose tissue. N Engl J Med. (2022) 386:768–79. doi: 10.1056/NEJMra2032804, PMID: 35196429

[28] Ringel AE Drijvers JM Baker GJ Catozzi A García-Cañaveras JC Gassaway BM. Obesity shapes metabolism in the tumor microenvironment to suppress anti-tumor immunity. Cell. (2020) 183:1848–66.e26. doi: 10.1016/j.cell.2020.11.009, PMID: 33301708 PMC8064125

[29] Conway JRW Dinç DD Follain G Paavolainen O Kaivola J Boström P. IGFBP2 secretion by mammary adipocytes limits breast cancer invasion. Sci Adv. (2023) 9:eadg1840. doi: 10.1126/sciadv.adg1840 37436978 PMC10337915

[30] Zheng R Zhang S Zeng H Wang S Sun K Chen R. Cancer incidence and mortality in China, 2016. J Natl Cancer Cent. (2022) 2:1–9. doi: 10.1016/j.jncc.2022.02.002, PMID: 39035212 PMC11256658

[31] Rock CL Thomson CA Sullivan KR Howe CL Kushi LH Caan BJ. American Cancer Society nutrition and physical activity guideline for cancer survivors. CA Cancer J Clin. (2022) 72:230–62. doi: 10.3322/caac.21719 35294043

[32] Graham JW. Missing data analysis: making it work in the real world. Annu Rev Psychol. (2009) 60:549–76. doi: 10.1146/annurev.psych.58.110405.085530, PMID: 18652544

