# Supplementary material for: The association between dietary creatine intake and cancer in U.S. adults: insights from NHANES 2007–2018
Source: Front Nutr. 2025 Jan 10;11:1460057. doi: 10.3389/fnut.2024.1460057 (PMC11757134; doi:10.3389/fnut.2024.1460057)
Supplement: Supplementary file 1 [file Table_1.docx]

Supplementary Material

# Supplementary Data

## Supplementary Table Multicollinearity Diagnosis for Continuous Variables

| Characteristic | Tolerance | VIF |
| --- | --- | --- |
| Average creatine intake(g) | .986 | 1.015 |
| Age(year) | .944 | 1.060 |
| Family income to poverty ratio | .954 | 1.048 |
| Blood cadmium(nmol/L) | .954 | 1.048 |
| Blood lead(nmol/L) | .924 | 1.082 |

## Supplementary Table 2 Bidirectional stepwise logistic regression analysis

| Variables | Univariate analysis | | | | |  | multivariable analysis | | | | |
| --- | --- | --- | --- | --- | --- | --- | --- | --- | --- | --- | --- |
|  | β | S.E | Z | P | OR (95%CI) |  | β | S.E | Z | P | OR (95%CI) |
| Average creatine intake(g) | -1.02 | 0.24 | -4.33 | <.001 | 0.36 (0.23 ~ 0.57) |  | -0.57 | 0.26 | -2.18 | 0.029 | 0.57 (0.34 ~ 0.94) |
| Sex |  |  |  |  |  |  |  |  |  |  |  |
| Female |  |  |  |  | 1.00 (Reference) |  |  |  |  |  | 1.00 (Reference) |
| Male | -0.03 | 0.04 | -0.65 | 0.518 | 0.97 (0.90 ~ 1.05) |  | -0.09 | 0.04 | -2.11 | 0.035 | 0.91 (0.83 ~ 0.99) |
| Age group |  |  |  |  |  |  |  |  |  |  |  |
| 20-51 years |  |  |  |  | 1.00 (Reference) |  |  |  |  |  | 1.00 (Reference) |
| 52-80 years | 2.02 | 0.06 | 35.88 | <.001 | 7.51 (6.72 ~ 8.38) |  | 1.80 | 0.06 | 29.93 | <.001 | 6.04 (5.37 ~ 6.79) |
| Race/ethnicityb, n(%) |  |  |  |  |  |  |  |  |  |  |  |
| Mexican American |  |  |  |  | 1.00 (Reference) |  |  |  |  |  | 1.00 (Reference) |
| Other Hispanic | 0.46 | 0.11 | 4.06 | <.001 | 1.59 (1.27 ~ 1.98) |  | 0.30 | 0.12 | 2.54 | 0.011 | 1.35 (1.07 ~ 1.69) |
| Non-Hispanic and Other race | 1.10 | 0.09 | 12.90 | <.001 | 3.00 (2.54 ~ 3.55) |  | 0.85 | 0.09 | 9.35 | <.001 | 2.35 (1.96 ~ 2.80) |
| Educationb, n(%) |  |  |  |  |  |  |  |  |  |  |  |
| <9th grade |  |  |  |  | 1.00 (Reference) |  |  |  |  |  | 1.00 (Reference) |
| 9–11th grade | -0.05 | 0.09 | -0.58 | 0.565 | 0.95 (0.79 ~ 1.14) |  | -0.04 | 0.10 | -0.40 | 0.686 | 0.96 (0.79 ~ 1.16) |
| High school diploma/GED | 0.06 | 0.08 | 0.73 | 0.468 | 1.06 (0.90 ~ 1.25) |  | 0.01 | 0.09 | 0.08 | 0.936 | 1.01 (0.84 ~ 1.20) |
| Some College/AA degree | 0.11 | 0.08 | 1.35 | 0.177 | 1.11 (0.95 ~ 1.30) |  | 0.15 | 0.09 | 1.68 | 0.092 | 1.16 (0.98 ~ 1.38) |
| ≥College graduate | 0.24 | 0.08 | 2.92 | 0.003 | 1.27 (1.08 ~ 1.49) |  | 0.25 | 0.10 | 2.65 | 0.008 | 1.29 (1.07 ~ 1.55) |
| Family income to poverty ratio | 0.11 | 0.01 | 8.41 | <.001 | 1.11 (1.09 ~ 1.14) |  | 0.06 | 0.02 | 3.55 | <.001 | 1.06 (1.03 ~ 1.09) |
| BMI |  |  |  |  |  |  |  |  |  |  |  |
| Normal |  |  |  |  | 1.00 (Reference) |  |  |  |  |  | 1.00 (Reference) |
| Underweight | 0.21 | 0.17 | 1.28 | 0.202 | 1.24 (0.89 ~ 1.71) |  | 0.32 | 0.18 | 1.80 | 0.072 | 1.37 (0.97 ~ 1.94) |
| Overweight | 0.12 | 0.06 | 2.17 | 0.030 | 1.13 (1.01 ~ 1.26) |  | -0.02 | 0.06 | -0.29 | 0.775 | 0.98 (0.87 ~ 1.11) |
| Obesity | -0.03 | 0.05 | -0.48 | 0.631 | 0.98 (0.88 ~ 1.08) |  | -0.19 | 0.06 | -3.32 | <.001 | 0.83 (0.74 ~ 0.93) |
| Blood cadmium(nmol/L) | 0.01 | 0.00 | 1.82 | 0.068 | 1.01 (1.00 ~ 1.02) |  |  |  |  |  |  |
| Blood lead(nmol/L) | 1.69 | 0.23 | 7.27 | <.001 | 5.43 (3.44 ~ 8.56) |  |  |  |  |  |  |
| Hypertensionb, n(%) |  |  |  |  |  |  |  |  |  |  |  |
| No |  |  |  |  | 1.00 (Reference) |  |  |  |  |  | 1.00 (Reference) |
| Yes | 0.95 | 0.04 | 22.98 | <.001 | 2.58 (2.38 ~ 2.80) |  | 0.36 | 0.05 | 7.90 | <.001 | 1.44 (1.31 ~ 1.58) |
| Drinking statusb, n(%) |  |  |  |  |  |  |  |  |  |  |  |
| No |  |  |  |  | 1.00 (Reference) |  |  |  |  |  |  |
| Yes | 0.23 | 0.07 | 3.25 | 0.001 | 1.26 (1.10 ~ 1.45) |  |  |  |  |  |  |
| NA | 0.03 | 0.07 | 0.44 | 0.659 | 1.03 (0.91 ~ 1.17) |  |  |  |  |  |  |
| Smoking statusb, n(%) |  |  |  |  |  |  |  |  |  |  |  |
| Never smoker |  |  |  |  | 1.00 (Reference) |  |  |  |  |  | 1.00 (Reference) |
| Current smoker | -0.05 | 0.06 | -0.78 | 0.434 | 0.95 (0.84 ~ 1.08) |  | 0.03 | 0.07 | 0.37 | 0.711 | 1.03 (0.90 ~ 1.17) |
| Former smoker | 0.77 | 0.04 | 17.41 | <.001 | 2.16 (1.98 ~ 2.36) |  | 0.41 | 0.05 | 8.54 | <.001 | 1.50 (1.37 ~ 1.65) |
| Diabetesb, n(%) |  |  |  |  |  |  |  |  |  |  |  |
| No |  |  |  |  | 1.00 (Reference) |  |  |  |  |  |  |
| Yes | 0.60 | 0.05 | 11.66 | <.001 | 1.82 (1.65 ~ 2.01) |  |  |  |  |  |  |
| Borderline | 0.65 | 0.11 | 5.94 | <.001 | 1.91 (1.54 ~ 2.36) |  |  |  |  |  |  |
| IPH9 |  |  |  |  |  |  |  |  |  |  |  |
| Mild |  |  |  |  | 1.00 (Reference) |  |  |  |  |  | 1.00 (Reference) |
| Moderate | 0.06 | 0.06 | 1.09 | 0.277 | 1.06 (0.95 ~ 1.19) |  | 0.10 | 0.06 | 1.67 | 0.095 | 1.11 (0.98 ~ 1.24) |
| Moderately Severe | 0.06 | 0.09 | 0.69 | 0.491 | 1.06 (0.89 ~ 1.27) |  | 0.15 | 0.10 | 1.59 | 0.112 | 1.17 (0.97 ~ 1.41) |
| Severe | 0.24 | 0.10 | 2.31 | 0.021 | 1.27 (1.04 ~ 1.56) |  | 0.33 | 0.11 | 2.96 | 0.003 | 1.39 (1.12 ~ 1.73) |
| Moderate exerciseb, n(%) |  |  |  |  |  |  |  |  |  |  |  |
| No |  |  |  |  | 1.00 (Reference) |  |  |  |  |  |  |
| Yes | -0.06 | 0.04 | -1.42 | 0.155 | 0.94 (0.87 ~ 1.02) |  |  |  |  |  |  |
